# Acoustofluidic localization of sparse particles on a piezoelectric resonant sensor for nanogram-scale mass measurements

**DOI:** 10.1038/s41378-021-00288-5

**Published:** 2021-08-13

**Authors:** Jingui Qian, Habiba Begum, Joshua E.-Y. Lee

**Affiliations:** 1 Department of Electrical Engineering, City University of Hong Kong, Kowloon, Hong Kong SAR China; 2 State Key Laboratory of Terahertz and Millimeter Waves, City University of Hong Kong, Kowloon, Hong Kong SAR China

**Keywords:** Chemistry, Electrical and electronic engineering

## Abstract

The ability to weigh microsubstances present in low concentrations is an important tool for environmental monitoring and chemical analysis. For instance, developing a rapid analysis platform that identifies the material type of microplastics in seawater would help evaluate the potential toxicity to marine organisms. In this study, we demonstrate the integration of two different techniques that bring together the functions of sparse particle localization and miniaturized mass sensing on a microelectromechanical system (MEMS) chip for enhanced detection and minimization of negative measurements. The droplet sample for analysis is loaded onto the MEMS chip containing a resonant mass sensor. Through the coupling of a surface acoustic wave (SAW) from a SAW transducer into the chip, the initially dispersed microparticles in the droplet are localized over the detection area of the MEMS sensor, which is only 200 µm wide. The accreted mass of the particles is then calibrated against the resulting shift in resonant frequency of the sensor. The SAW device and MEMS chip are detachable after use, allowing the reuse of the SAW device part of the setup instead of the disposal of both parts. Our platform maintains the strengths of noncontact and label-free dual-chip acoustofluidic devices, demonstrating for the first time an integrated microparticle manipulation and real-time mass measurement platform useful for the analysis of sparse microsubstances.

## Introduction

Microplastics (MPs), such as polystyrene (PS) microbeads and polyvinyl chloride fragments, have long degradation lifespans. Their persistence in water bodies and harm to aquatic organisms and ecosystems make them a significant environmental concern^1^. While there are existing technologies for collecting and analyzing large MPs (diameters ≥20–50 μm) for detection and processing^2^, tools targeting smaller MPs of a few microns are lacking^3^. Such small MPs have profound long-term toxic effects on marine life, such as phytoplankton, zooplankton, shellfish, and corals^4^. As such, the dangers posed by even a small trace of MPs cannot be underestimated. However, the rarity of MPs in small traces adds further requirements to their handling and analysis, such as centrifugation prior to analysis. A smart label-free tool that integrates centrifugation and analytical functions would therefore be beneficial.

Regarding analytical tools, there has long been interest in applying microelectromechanical system (MEMS) resonators for mass-based sensing given their high sensitivity, low volume sample requirements, and digitized readouts^5,6^. Thin-film piezoelectric-on-silicon (TPoS) MEMS resonators comprise a thin piezoelectric layer (e.g., aluminum nitride (AlN)) deposited on a thicker silicon carrier and have been of interest for liquid-phase measurements^7,8^. The working principle of a TPoS MEMS resonator starts with the actuation the resonator through the application of a harmonic voltage signal across the piezoelectric transducer. The resonator vibrates due to the reverse piezoelectric effect. Conversely, the mechanical strain associated with the vibration is converted to an output current due to the piezoelectric effect. The TPoS structure is well suited for weighing MPs due to the benefits of the more efficient electromechanical coupling of by piezoelectric transduction and the addition of a silicon carrier, slightly enhancing the quality factor (Q-factor)^9^.

While there have been many reports on miniaturizing MEMS resonators to enhance sensitivity^10^, there is a critical gap in regard to techniques for attracting dispersed MPs to highly miniaturized sensing devices. More specifically, a MEMS resonator may have a detection area no larger than several hundred microns, but the diameter of a 4 μL sample droplet exceeds 2 mm. Hence, in the case of a sample droplet with a small trace of MPs, it would be rather common for there to be no MPs on the resonator and thus for no measurements to be returned despite several attempts. What is critically missing is a practical means of agilely gathering extensively scattered MPs and localizing them on a sensing device.

Recently, acoustic tweezers that use surface acoustic waves (SAWs) to handle particles and biological matter in complex fluids have been widely studied^11–15^. This approach has the benefits of being label-free, consuming relatively low amounts of power, and being contactless in nature^16^. Ghulam et al. proposed a mechanism for concentrating particles inside a sessile droplet by tuning the actuation frequency and particle diameter^17^. Yannyk et al. employed SAWs to realize rare-cell enrichment and separation in a fingerpick-sized drop of blood^18^. We have previously reported a plug-and-play acoustofluidic device for droplet microcentrifugation that enables particle focusing and isolation on a surface-micromachined silicon (SMS) chip^19^. Such a proposed dual-chip strategy facilitates the reusability of the SAW device while retaining the benefits of acoustofluidic methods and is applicable to various microparticles with diverse densities and sizes^20^. Some researchers have also described the conversion of Lamb waves to Rayleigh waves on an SMS superstrate (e.g., MEMS chip) or a superstrate with a single metal thin film deposited, which could be more efficient for particle manipulation^21,22^.

First, this work applies acoustofluidic microcentrifugation techniques^23–25^ to a MEMS sensor chip to combine the capabilities of sparse particle localization and nanogram-scale mass detection in a plug-and-play dual-chip format with one chip (SAW transducer) to generate acoustic waves that couple into a second chip (MEMS chip) where the mass of the MPs is measured. We propose such a dual-chip approach as a more viable solution than a fully integrated solution in terms of cost and fabrication complexity. In the proposed dual-chip approach, while the MEMS chip may have to be disposed of after use, the SAW transducer can be reused, as the two parts can be detached without damage. We describe the considerations in the design of the TPoS MEMS resonator regarding compatibility with an acoustofluidic device setup. The application of acoustofluidic forces to a MEMS chip requires that the droplet on the top surface of the MEMS device be completely sealed from the bottom of the chip in contact with the SAW devices via a coupling gel. In addition, to ensure that the MEMS chip can be peeled off of the SAW device after each use, the anchoring of the thin diaphragm structure in the MEMS chip must be sufficiently robust to avoid breakage at the weakest links. Within these constraints, the Q-factor of the resonator must be sufficiently high to resolve frequency shifts from the addition of a few MPs. Next, we describe a plug-and-play method to combine the SAW device and TPoS MEMS chip via a suitable ultrasonic couplant. We then investigate the particle localization performance in a sessile droplet on the MEMS chip with respect to different particle diameters. Finally, we demonstrate the capability of localizing initially dispersed sparse particles on the TPoS MEMS resonator, calibrating the measured resonant frequency shift to the accreted mass. This work marks a first step toward directly manipulating microparticles on a MEMS resonant sensor in a sessile droplet for real-time measurements.

## Working mechanism

**Fig. 1:**
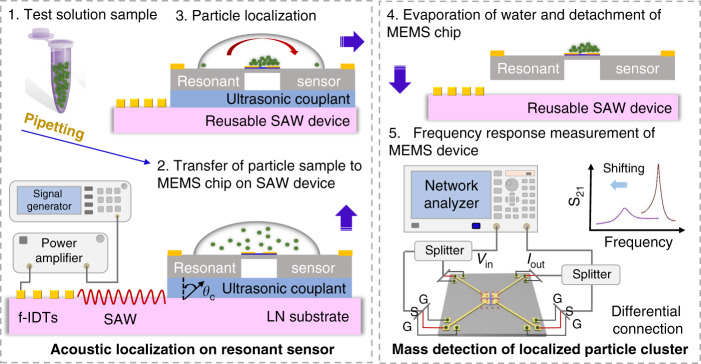
Methodology and workflow for acoustofluidic particle localization over a TPoS resonant sensor followed by mass detection of the localized particle cluster. (1) A water sample containing micro-objects (e.g., seawater or an insoluble chemical solution) is to be collected in tubes. (2) The MEMS chip is mounted on a SAW device with an ultrasonic couplant at the interface. A small test sample (μL) containing the MPs is then pipetted onto the MEMS chip. (3) SAWs generated from the LiNbO_3_ (LN) piezoelectric substrate are transmitted into the MEMS chip (by diffraction at the Rayleigh angle θ_c_) to drive droplet microcentrifugation, resulting in the localization of particles on the resonant sensor. (4) The droplet is left to evaporate within several seconds, leaving behind the micro-objects on the TPoS resonant sensor. The MEMS chip can be detached from the LN substrate without damaging either device. (5) This TPoS MEMS device was probed using a fully differential configuration with electrical transmission (S_21_) measured using a network analyzer and a pair of radiofrequency (RF) power splitters. The mass of localized micro-objects was calibrated to the measured shift in the resonant frequency before and after adding the localized particles.

As delineated in Fig. 1, there are two major experimental phases involved in the setup: (1) acoustofluidic particle localization in a sessile droplet on the MEMS chip and (2) mass measurement of the localized particle cluster by measuring the frequency shifting of the MEMS resonant sensor. First, the solution of PS particles is diluted with deionized (DI) water in a tube to mimic a collected sample with sparse MPs. Next, the MEMS chip is mounted on the SAW device with the colloidal ultrasonic couplant providing a temporary adhesive, as mentioned in the Materials and methods section. After a sample droplet is pipetted onto the desired position on the MEMS chip (e.g., directly on top of the resonant sensor), a Rayleigh acoustic wave is generated by inputting an RF signal across the fanned interdigitated transducers (f-IDTs). With an f-IDT design, the period between electrodes varies across the aperture of the IDT. This creates a narrow acoustic beam at different positions along the aperture based on the actuation frequency applied. As such, the frequency is tuned to position the path of the beam to be off the center of the sessile droplet to create an angular momentum inside the droplet. The acoustic wave coupled into the MEMS chip (e.g., superstrate) interacts with the periphery of the sessile droplet and is refracted in the droplet. Details on acoustic wave propagation and the underlying mechanism of coupling waves between the different domains involved in such a sandwich-structured device have previously been extensively described^22,26^. The backside cavity and surface multilayers on the MEMS resonator chip have little effect on the effectiveness of the acoustic localization of particles in a droplet (see S1 of the Supplementary information). The generated acoustic streaming force provides a rotational force component, while the acoustic radiation force provides a radial component that pushes the particles to the center of the droplet^19^.

The key to realizing a dual-chip setup is the stable adhesion and ease of detachment of the two parts. As shown in steps 3–4 of Fig. 1, the ability to detach the two parts while ensuring suitably high acoustic transmission efficiency is enabled by the chosen ultrasonic couplant. A uniform coating of the couplant provides reliable and reproducible acoustic particle localization performance each time when changing between MEMS chips. The selected colloidal gel dissolves quickly in DI water and 75% ethanol, which enables the seamless detachment of the two chips, with no damage to either chip during the detachment process, making the SAW device reusable. Note that the MEMS chip can also be reused after removing PS particles approximately three times.

With the particles localized over the resonant sensor using acoustic microcentrifugation, the droplet on the MEMS chip is left to evaporate within several seconds. The MEMS chip is then detached from the SAW device without damage and transferred to the probe station for electrical probing. The sensitivity of the resonant sensor to the loading of microbeads on the device in terms of resonant frequency shift (*Δf*) as a linear function of the added mass is given by the first-order approximation in the Taylor series expansion,

1 $${\Delta} f = - \frac{1}{2}f(\frac{{{\Delta} k}}{k} - \frac{{{\Delta} m}}{m})$$

where *f* is the nominal unperturbed resonant frequency, *m* is the dynamic mass, and *k* is the stiffness, all with respect to the MEMS resonator. *Δm* and *Δk* are the changes in mass and stiffness, respectively, as a result of particle localization on the top of the MEMS resonant sensor.

## Results and discussion

### Structural design, fabrication, treatment, and assembly of the acoustofluidic MEMS-microbalance platform

**Fig. 2:**
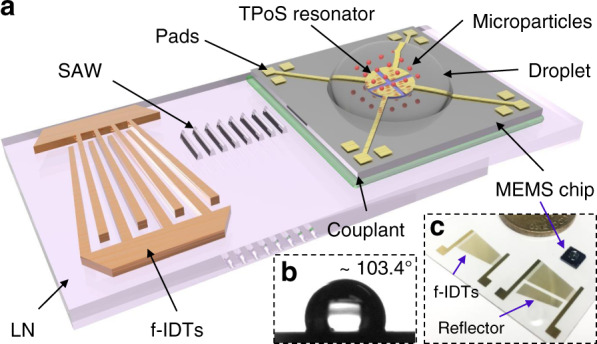
Description of the acoustofluidic MEMS-microbalance platform. **a** Schematic diagram of the dual-chip SAW transducer and TPoS MEMS chip acoustofluidic and sensing platform. The TPoS MEMS chip is mounted on the LN SAW device with an ultrasonic couplant gel interface. A μL volume of sample droplet containing microparticles is loaded on top of the MEMS resonator. **b** Contact angle of a 2 μL droplet on the treated surface of the MEMS chip to verify the desired hydrophobicity of the surface. **c** Optical image of the dual-chip acoustofluidic and sensing device, of which the size is referenced to a 1 HKD coin.

Fig. 2a depicts a perspective view schematic of the dual-chip acoustofluidic SAW transducer and MEMS chip hybrid device. To realize sessile droplet microcentrifugation to localize scattered particles at the center of the droplet (where the MEMS resonator is located), a spatially asymmetric acoustic field is generated on the SAW transducer (substrate) and coupled into the MEMS chip (superstrate). Three methods for generating asymmetric fields have been reported, the use of f-IDTs in SAW transducer design^19^, the employment of a frequency selective phononic crystal etched into a superstrate^18^, and the use of nonfrequency selective periodic structures patterned in a superstrate^27^. For simplicity of implementation, f-IDTs are utilized in this work. The position of the SAW propagation beam along the aperture is adjusted by tuning the excitation frequency to conveniently generate an asymmetric acoustic field after several attempts. For efficient droplet microcentrifugation while avoiding excessive excitation RF signal power, the actuation frequency should lie within the range from 10 to 20 MHz^17^. To operate within this frequency range, the graded periods of the f-IDTs are designed to range from 100 to 200 μm, corresponding to a frequency range of 9.975–19.95 MHz. To ensure that the area of the MEMS chip is fully confined within the acoustic field generated over the full range of frequencies, the aperture/length of the f-IDTs is designed to be 11 mm, which is double the side length of the MEMS chip. The SAW device design also ensures repeatable outcomes in particle localization with tolerance to some variation in different MEMS chips in terms of chip size and attachment location during the assembly process. To enhance unidirectional propagation of the SAW, IDT reflectors are added to the exterior side of the f-IDTs. The SAW generated on the LN substrate couples into the MEMS chip to drive microcentrifugation. Finally, after acoustic treatment, the initially scattered particles in the sample droplet (pipetted onto the MEMS chip) are localized directly on top of the MEMS resonant sensor.

In the equilibrium state, a high contact angle (≥90°) between the droplet and the surface results in a complete hemisphere that helps increase the efficiency of acoustic manipulation (e.g., pushing particles toward the center of the droplet)^27–29^. As such, the surface of the MEMS chip is treated to deliver a similar degree of hydrophobicity (contact angle of 103.4°), as captured in Fig. 2b. An optical image of the fabricated and assembled platform is pictured in Fig. 2c, where the size is referenced to a 1 HKD coin. As mentioned above, the bonding between the MEMS chip and SAW transducer is impermanent, allowing repeated detachment of the MEMS chip from the SAW transducer without damaging the reusable SAW device.

### Mode simulation and structure design of the TPoS resonant sensor

Not all MEMS resonator designs are compatible with a dual-chip acoustofluidic setup. Certain design constraints must be considered in the conceptualization of the MEMS resonator. For instance, the top surface of the MEMS resonator must be sealed from ultrasonic couplant gel at the bottom surface of the MEMS chip. This is because during acoustic actuation, liquid from the droplet on the top side of the MEMS chip flows through any path that connects the top side and bottom side of the chip (see S2 of the Supplementary information). In cases where the resonator is released from the handle wafer by deep etching of a back cavity through the handle wafer, etching gaps through the thin resonator device layer inevitably introduce a connecting path between the top side and bottom side of the MEMS chip through which fluids can move. Under acoustic action, the droplet (only a few microliters) quickly flows from the top side to the bottom of the MEMS chip, thereby interrupting the process of acoustic particle localization. In such cases, the resonator device layer must be fully sealed to be compatible with an acoustofluidic setup. This is the case for the fabrication process we use here. The narrow side supports typical of contour mode resonators are also not preferred for an acoustofluidic setup, as they introduce points of weakness, which from our experiments with other TPoS MEMS resonators poses practical problems of breakage in the process of repeated use. Based on the above constraints, we choose a circular diaphragm resonator fully clamped around its entire circumference with a diameter of 230 µm (i.e., no side gaps). Given that the diaphragm is clamped around its entire circumference, the resonator is designed to vibrate in transverse plate bending mode. As a high Q-factor is beneficial for the measurement of the resonance, the electrode layout is designed to preferentially excite the higher-order transverse (2, 0) mode as opposed to lower-order modes that typically offer a lower Q-factor. The enhanced Q-factor from transducing the higher-order transverse (2, 0) mode proves critical to observing the shift in the resonant peak associated with adding a nanogram of microparticles after acoustic microcentrifugation.

**Fig. 3:**
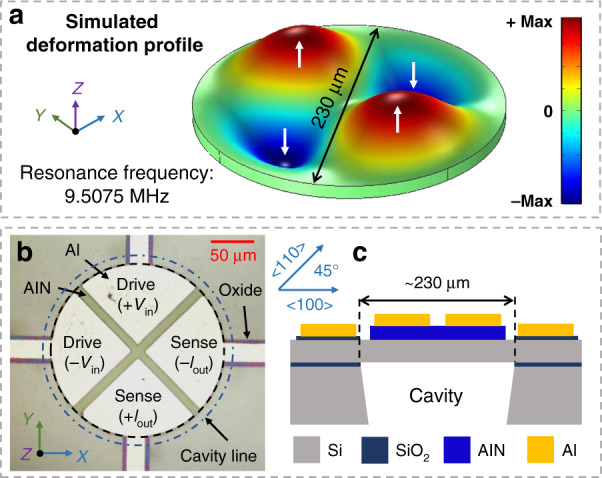
Description of the MEMS TPoS resonant sensor. **a** Simulated deformation profile for the transverse (2, 0) mode excited in a circular diaphragm (diameter of 230 μm) using COMSOL Multiphysics 5.5, which shows the distribution of the absolute (i.e., vector sum or modulus) displacement. **b** Micrograph showing the top side of the fabricated circular diaphragm resonator and transducer electrode layout with the transduction ports labeled based on differential driving and sensing. **c** Side view schematic of the TPoS circular diaphragm resonator showing the constituent thin film layers and through-wafer back cavity.

We simulate the deformation profile of the transverse (2, 0) mode by finite elements using COMSOL Multiphysics 5.5, as shown in Fig. 3a, where the color contours represent the vertical displacement distribution. In the model, the diaphragm is fully clamped around its circumference. As shown in Fig. 3a, the transverse (2, 0) mode is characterized by a complementary pair of peaks and troughs. The simulated frequency for a 230-μm-diameter silicon diaphragm resonating in the transverse (2, 0) mode is 9.5075 MHz. As shown in Fig. 3b, the top side of the diaphragm resonator is defined by four transducer electrodes that coincide with the regions of peak transverse displacements. The transverse (2, 0) mode is actuated by a differential pair of input RF voltages (±V_in_) applied to two adjacent transducer electrodes and electrically detected by the other two adjacent transducer electrodes as a differential pair (±I_out_). The silicon device layer is surface doped and acts as the bottom ground electrode. This fully differential transduction configuration helps to reduce the effects of parasitic feedthrough between the input and output ports.

As depicted in the schematic of Fig. 3c, the fabricated TPoS AlN-on-Si resonant sensor primarily comprises a 10-μm-thick silicon device layer, 0.5-μm-thick AlN piezoelectric thin film, and 1-μm-thick Al layer for the top metal electrodes. The diaphragm disk is fully clamped to the Si substrate. Note that the specified diaphragm diameter of 230 µm is an estimate based on process monitoring structures that indicate an overetching of 20–30 µm from the back side through-wafer deep reactive ion etching (DRIE). The transducer electrodes have a sectorial radius of approximately 100 µm.

### Electrical characterization of the SAW transducer

**Fig. 4:**
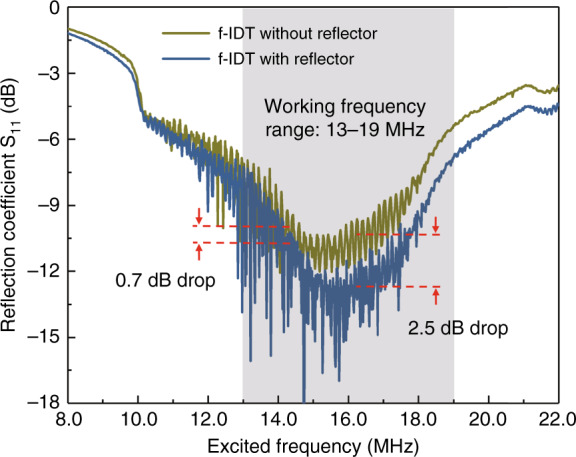
Comparison of the measured reflections (S_11_) of two fabricated SAW devices with the same f-IDT parameters where one has a reflector on the exterior side of the f-IDTs and the other does not. The shaded region indicates the working range of frequencies used for the particle localization experiments.

Prior to performing the particle localization experiments, we measure the electrical reflection (S_11_) of the fabricated SAW transducers by comparing the results from SAW devices with and without reflectors behind the f-IDTs (Fig. 4). The results verify the usefulness of the reflector in reducing acoustic wave backhaul and thereby enhancing propagation in the forward direction. Within the range of frequencies used in this work for microcentrifugation (13–19 MHz), Fig. 4 shows a reduction in the reflection by 0.7–2.5 dB with the addition of reflectors on the exterior side of the f-IDTs. The increased transmission within the intended working frequency range provides enhanced efficiency in particle localization on the MEMS chip.

### Acoustic localization of microparticles on a MEMS chip

**Fig. 5:**
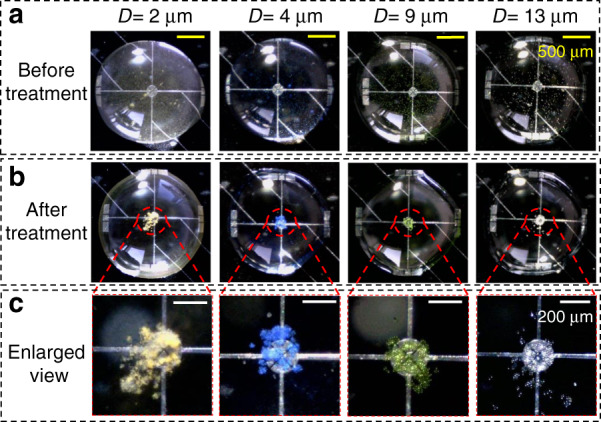
Acoustic microcentrifugation in a water droplet to localize particles on a MEMS resonator chip with a back cavity tested for different diameters (*D*) of PS particles. **a** Initial state of particles in the same volume of water droplet (4 μL) prior to acoustic actuation, where the particles are randomly distributed. **b** Clustering of particles to the center of the sessile droplet after acoustic treatment using an input RF signal with 33 dBm (±2 dBm) input power. **c** Enlarged view of the particle cluster above the TPoS MEMS resonator for the different particle diameters (differentiated according to dye color) tested.

While there are many reports on using acoustic waves to manipulate the particles in a sessile droplet on bare superstrates^19^ or even superstrates with multilayered surface thin films^22^, the efficacy of acoustic microcentrifugation on a superstrate with a through-wafer back cavity has yet to be tested. As such, we investigate the particle localization performance in a water droplet on a MEMS chip with a TPoS resonant sensor using PS microbeads with different diameters. For these tests, dyed PS microbeads are diluted in DI water to obtain a concentration of 0.125 mg/mL. We choose such a moderately high particle concentration to increase the visibility of the particles when examining the experimental results. The test results for four different PS bead diameters are recorded in Fig. 5. In each of the four tests, the same droplet volume of 4 μL is pipetted on the MEMS chip at approximately the same position: on the MEMS resonator in the center of the chip. Figure 5a shows the state of the particles, distributed randomly in the sessile water droplet, prior to applying the acoustic wave. The diameters of the PS beads are differentiated by dye color: 2 μm (yellow), 4 μm (blue), 9 μm (green), and 13 μm (white). After acoustic treatment for approximately 10–20 s with an input RF power of 33 dBm (±2 dBm), the PS microbeads cluster around the center of the droplet (Fig. 5b, most of which are above the TPoS resonant sensor, as seen from the enlarged views displayed in Fig. 5c). As such, we successfully demonstrate the ability of microcentrifugation to localize particles on a MEMS chip with a through-wafer backside cavity structure. The benefits of this capability are illustrated in the results in the following section. Typically, the particles remain in the same position in the liquid droplet after switching off the acoustic wave (e.g., at the bottom center of the droplet after microcentrifugation)^27,28^. The surface adhesion of PS particles to the underlying surface (i.e., the resonator top surface in this case) also helps to prevent the localized particles from ambulating and dispersing outside the resonator after off the acoustic field is switched off.

### Nanogram mass detection of localized particle clusters

**Fig. 6:**
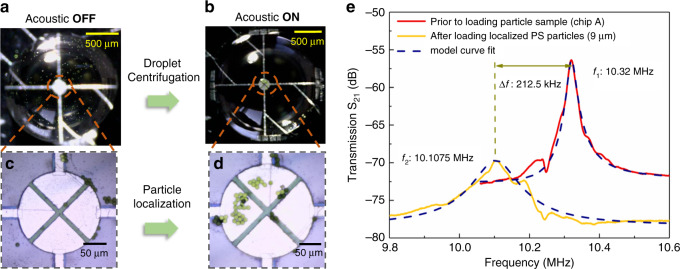
Demonstration of the fusion of sparse particle localization and miniaturized mass sensing. **a**, **b** Localization of scattered sparse PS beads on the small TPoS resonator area by acoustic actuation. **c**, **d** Closeup view of the resonator area prior to and after acoustic localization. After the water droplet evaporates, none of the particles are found on the resonator, while almost all the particles are localized on the resonator area. **e** Comparison of the measured S_21_ of the TPoS resonator (Chip A) in air with no particles added and after acoustically localized PS particles (9 μm in diameter) are loaded on the resonant sensor. The addition of the localized particles results in a clear shift in the resonant frequency along with an attenuation and broadening of the peak. The broken lines are model curve fits based on a series resonant equivalent circuit model that describes the lumped electrical characteristics of the TPoS resonator (Fig. S3 of the Supplementary information).

To mimic the scenario of detecting sparse particles present in low concentrations, such as MPs present in seawater, we prepare a suspension sample of PS microbeads at a low concentration by diluting the initial solution of PS microbeads in DI water down to a concentration of 0.025 mg/mL. Figure 6 compares the distribution of 9-μm-diameter PS beads on the TPoS resonator sensing area before and after acoustic treatment in a typical test run. Prior to the application of acoustic actuation, the particles are clearly visible in different parts of the water droplet, as shown in Fig. 6a. However, a closer look at the resonator area, depicted in Fig. 6c, reveals that none of the particles are on the resonator area after evaporation. Such a situation is very common in the multiple tests conducted given the sparsity of particles present. Measuring the frequency response of the device in such instances would thus return a negative result without the means to control how the particles are located relative to the TPoS resonant sensor. Trying to obtain one or a few particles on the resonator area relies on multiple trial-and-error attempts given that the distribution of the particles is random. Acoustic microcentrifugation provides us with the needed control to localize the sparse number of scattered particles at the desired location. As shown in Fig. 6b, applying acoustic microcentrifugation drives the sparse number of particles onto the resonator area, as indicated by the absence of particles in the vast space around the center of the droplet. The closeup view in Fig. 6d shows that the particles are localized on the resonator, where their mass can be measured by the TPoS resonant sensor.

The MEMS chip (labeled Chip A) is then detached from the SAW transducer and placed on a probe station (RF GSG probes) for electrical characterization of the frequency response. The frequency response of the MEMS resonator is measured under atmospheric conditions after the water droplet evaporates using a network analyzer, where we apply an RF input power of 0 dBm. Fig. 6e compares the frequency response (S_21_) of the TPoS resonator with the localized PS beads loaded against the frequency response prior to adding the PS beads. Fig. 6e shows a clear downshift from the preloaded unperturbed resonant frequency of 10.32 MHz by 212.5 kHz (i.e., 2.06%) due to the accretion of approximately 42 PS particles (diameter of 9 μm). In addition to causing a resonant frequency shift, accretion of the particles causes a broadening and attenuation of the peak, which only occurs when there are particles on the device. In instances where the particles are dispersed on the chip but not located on the resonator, there is no broadening or attenuation of the peak.

**Fig. 7:**
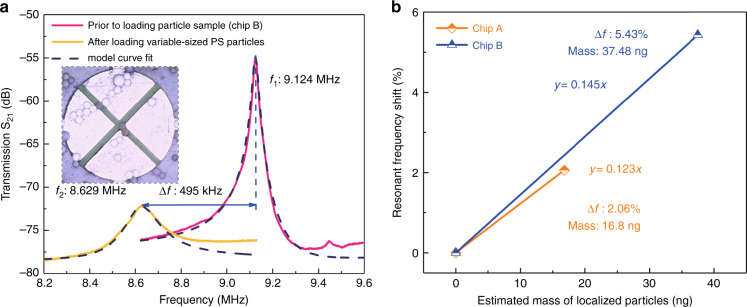
Mass detection of acoustically localized particles of various diameters on a TPoS resonant sensor and measurement of mass sensitivity. **a** Comparison of the measured electrical transmission (S_21_) of a TPoS resonator (Chip B, same design as Chip A) in air with no particles added and after acoustically localized PS particles of a range of sizes are added. **b** Comparison between Chip A and Chip B of the measured frequency shift normalized over the nominal frequency as a function of the mass of the localized PS particles accreted on each TPoS resonator.

The experiments described thus far are all based on particles with the same size. However, in actual situations, the particle diameters in real samples would likely exhibit a range of values. To demonstrate the reproducibility and consistency of the test results, we use another chip sample (labeled here as Chip B) of the same TPoS resonator design to measure the mass of PS microbeads with a range of diameters: 12–22 μm). The same procedure involving acoustic actuation for particle localization, MEMS chip detachment, and MEMS device characterization is employed with Chip B but with a solution sample containing PS beads representing a range of diameters. Figure 7a compares the measured frequency response of Chip B with the localized PS particles loaded against the preloaded frequency response. The inset shows a micrograph of the TPoS resonator area with PS microbeads of different sizes (12 μm: 3, 13 μm: 2, 14 μm: 16, 22 μm: 1; and there is half a 20 μm bead on the resonator). As in the case of Chip A, accretion of the particles on the resonator causes a downshift in the resonant frequency from the preloaded unperturbed value of 9.124 MHz by approximately 495 kHz (5.43%). The slope of the frequency shift to the loaded mass calibration graph corresponds to the sensitivity of the resonant mass sensor. For the cases of Chip A and Chip B, whether the sizes of particles are uniform or vary across a range, Fig. 7b shows almost the same sensitivity.

The preloaded unperturbed resonant frequencies of Chip A and Chip B are slightly different due to the variations in the slope angle from the through-wafer DRIE of the back cavity. The observed difference in the frequency (10%) falls within the expected variation in the diaphragm size due to the DRIE overetch (10 μm, corresponding to 5% of the diaphragm diameter) given the inverse square law relation between the resonant frequency scale diaphragm diameter. The extracted lumped parameters of the TPoS resonators on Chip A and Chip B based on model curve fits are summarized in S4 of the Supplementary information. In the case of almost all MEMS resonators, the nonuniformity of the particle distribution would theoretically affect the sensitivity. However, in practice, Fig. 7b shows that the nonuniformity results only in a slight difference in sensitivity. This is due in part to the displacement profile of the transverse (2, 0) mode design that comprises four quadrants with the same effective sensitivity, thereby increasing the effective capture area among several particles distributed over the resonator. In addition, as shown in S5 of the Supplementary information, temperature sensitivity measurements over the range of 298–308 K show a frequency shift of 300 ppm (i.e., 30 ppm/K). This value of the temperature coefficient of frequency is consistent with silicon-based resonators. Assuming a maximum variation of 2 °C between measurements under normal lab conditions, the effect of variations in temperature is insignificant for the magnitude of the frequency shift due to the addition of nanograms of particles reported here.

## Conclusions

This work describes the complementary fusion of two functions in a plug-and-play dual-chip format: (1) acoustic manipulation to focus particles dispersed in a droplet on a chip and (2) mass sensing with a MEMS resonator on the chip. With the two functions combined, sparse particles dispersed in a droplet are localized on the MEMS sensor with high precision, thereby enhancing the effective sensitivity of measurements while largely eliminating the occurrence of negative measurements (i.e., no particles are found on the MEMS resonator). Without a technique to manipulate the particles on the MEMS chip, multiple trial-and-error attempts must be used to randomly land the sparse number of particles in the sample droplet on the resonator. We test and compare experiments with particles of the same diameter and a variety of diameters, showing that the results are consistent. As a plug-and-play dual-chip setup, the SAW device and MEMS chip are detachable on demand, making the SAW device reusable. Fusing two functions together on a unified platform provides the ability to detect low concentrations of microsubstances such as MPs, which could be a powerful tool for environmental monitoring and chemical analysis for analyzing MPs in seawater and insoluble chemical solutions.

## Materials and methods

### Fabrication of acoustofluidic MEMS-microbalance platform

There were three major steps for preparing the dual-chip platform: (1) fabricating the reusable SAW device, (2) fabricating the TPoS MEMS sensor chip, and (3) assembling the MEMS chip on the SAW device and temporary adhesion thereof by a colloidal couplant. For the SAW device, standard photolithography was used to pattern f-IDTs on a 128° Y-cut X-propagation LiNbO_3_ wafer (PWLN-431232, Pmoptics, USA), followed by deposition of chrome (50 Å) and gold (600 Å) by thermal evaporation (Denton Vacuum, USA) and a lift-off process.

Next, the TPoS MEMS sensor chip was fabricated using the PiezoMUMPs^TM^ process by MEMSCAP^®^^30^. The process starts with a silicon-on-insulator wafer that includes a 400-μm-thick handle layer, a 1-μm-thick buried oxide layer, and a 10-μm-thick silicon device layer. A 200-nm-thick thermal oxide is grown and patterned by wet etching for insulation. This is followed by sputtering 0.5 μm of piezoelectric AlN that is then patterned and wet-etched. A metal stack of Cr/Al (20 nm/1000 nm) is deposited and patterned to define the top electrodes and the contact pads. Although the process offers RIE of the thermal oxide and DRIE of the silicon device layer to define features in the silicon device layer, this step was not utilized to fabricate the MEMS device described in this work. Finally, a back cavity is etched through the handling layer by DRIE followed by buffered hydrofluoric wet etching of the buried oxide layer to release the diaphragm structure defined by the silicon device layer. Each MEMS chip had a die size of 5.4 mm by 5.4 mm and contained a single TPoS MEMS resonant sensor at the center.

Prior to each acoustic localization experiment, the MEMS chip was mounted on the SAW device via a layer of colloidal ultrasonic couplant (Guanggong, China). The selected coupling gel offers long-term stability and efficient acoustic transmission between two surfaces^19^. After each experiment, the MEMS chip could be detached from the SAW substrate, making the SAW device reusable. The water-soluble ultrasonic gel was easily removed by DI water or 75% ethanol solution.

### Hydrophobic treatment and sample preparation

A slightly obtuse contact angle between the sessile droplet and MEMS chip surface helped to increase the efficiency of particle localization and prevented the droplet from spreading out after acoustic actuation. The surface of the MEMS chip was treated to obtain slight hydrophobicity (contact angle of 103.4°). The treatment solution comprised 50 μL of trichloro (1H, 1H, 2H, 2H-perfluorooctyl) silane (Sigma-Aldrich) diluted in 50 mL of n-hexane. This was followed by a salinization process for 15 min at 120 °C after O_2_ plasma treatment for 5 min at a power of 18 W.

PS microbeads were employed to characterize the acoustofluidic localization performance and mimic sparse microsubstances in an aqueous solution for measurement on the MEMS resonant sensor. Dyed PS particles (Da’e Scientific, Tianjin, China) were diluted in DI water to obtain two concentrations (high: 0.125 mg/mL, low: 0.025 mg/mL). The density of the PS particles was 1.05 gcm^–3^. We tested for four different particle diameters: 2, 4, 9, and 13 μm.

### Experimental setup and measurement system

The SAW device and TPoS resonant sensor were measured using a network analyzer (Agilent E5061A). A vector signal generator (Agilent MXG 8644B) in conjunction with a power amplifier (Mini Circuits ZHL-5W-1, 5-500 MHz) provided the input RF signal to the SAW transducer. During the acoustofluidic experiments, the assembled platform was placed on the stage of a digital microscope (AM7515MZTL, Dino-Lite, Taiwan) to record the particle behavior in a sessile droplet. To electrically probe the MEMS resonant sensor, the MEMS chip was first detached from the SAW device and transferred to a probe station (Janis model ST-500-2-2TX-2MW, USA). Two RF power splitters (Mini Circuits ZFSCJ-2-1-S+, 1–500 MHz) were used to realize a fully differential (differential input, differential output) connection. A sample droplet of PS microbeads (approximately 4 μL) was pipetted (Eppendorf, 20 μL, Germany) on top of the TPoS resonant sensor in the MEMS chip. The contact angle was measured by a drop shape analyzer (ARUSS DSA100, Germany). A passive aluminum heat sink was placed under the SAW device to dissipate the heat generated during acoustic actuation.

## Supplementary information

Supplementary information
