# Supplementary material for: Acoustofluidic localization of sparse particles on a piezoelectric resonant sensor for nanogram-scale mass measurements
Source: Microsyst Nanoeng. 2021 Aug 13;7:61. doi: 10.1038/s41378-021-00288-5 (PMC8433202; doi:10.1038/s41378-021-00288-5)
Supplement: Supplementary file 1 — Supplementary information [file 41378_2021_288_MOESM1_ESM.docx]

**Supplementary information**

**Acoustofluidic** **localization of sparse particles on a piezoelectric resonant sensor for nanogram-scale mass measurements**

Jingui Qian^1^, Habiba Begum^1^ and Joshua E.-Y. Lee^*, 1, 2^

^1^Department of Electrical Engineering, City University of Hong Kong, Kowloon, Hong Kong SAR, China

^2^State Key Laboratory of Terahertz and Millimeter Waves, City University of Hong Kong, Kowloon, Hong Kong SAR, China

**Corresponding author:** Joshua E.-Y. Lee

E-mail addresses: [josh.lee@cityu.edu.hk](mailto:josh.lee@cityu.edu.hk) Tel. (+852) 3442 9897

**S1 Additional discussion on the effect of the back cavity on acoustofluidic localization**


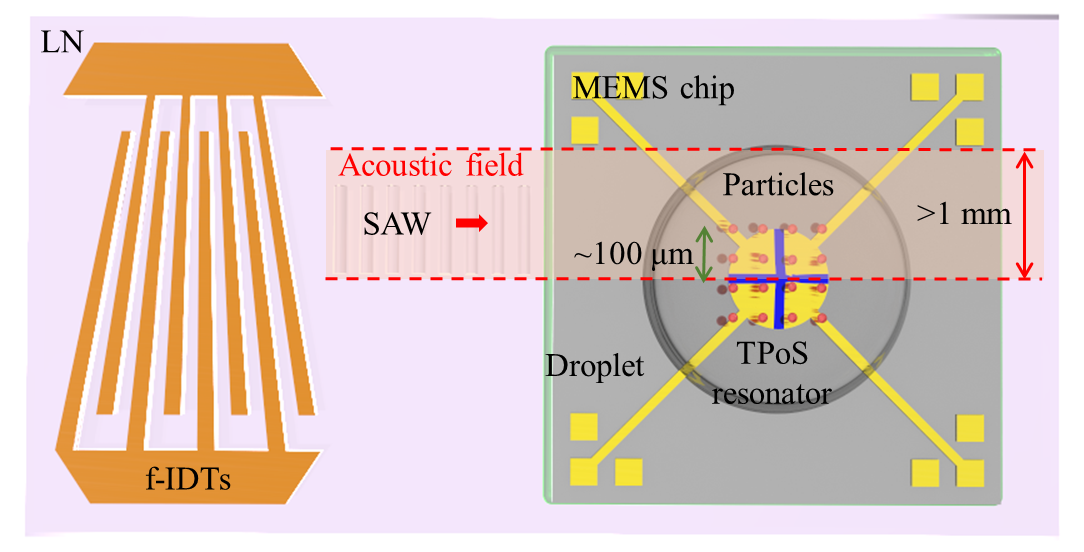


Figure S1. Front view of the proposed device shows the narrow beam path of the acoustic wave exposed to half of the droplet. Note: not drawn to scale.

In addition to existing experiments based on plain silicon superstrates, we have previously shown in [1] that dual-chip micro-centrifugation can be realized just as efficiently in superstrates with surface micromachined multi-layered microstructures. In this paper, we go a step further by introducing a back cavity beneath the MEMS resonator as a backside release feature. We have found that introducing the back cavity has little effect on the localizing particles. As shown in Fig. S1, the diameter of the back cavity is about 230 μm, which is much smaller than the diameter of a 4 μL sessile droplet (> 2 mm). Typically, a narrow beam acoustic field was generated by the f-IDT to drive acoustofluidic localization. To realize particle localization, only up to half of the water droplet needs to be exposed to the incident acoustic field. Therefore, even the absence of waves locally at the resonator does not affect the acoustic localization performance that is driven by waves propagating in other parts of the MEMS chip as confirmed by Fig. 5.

**S2 Two kind of designs of TPoS resonator with and without etching gaps**

**
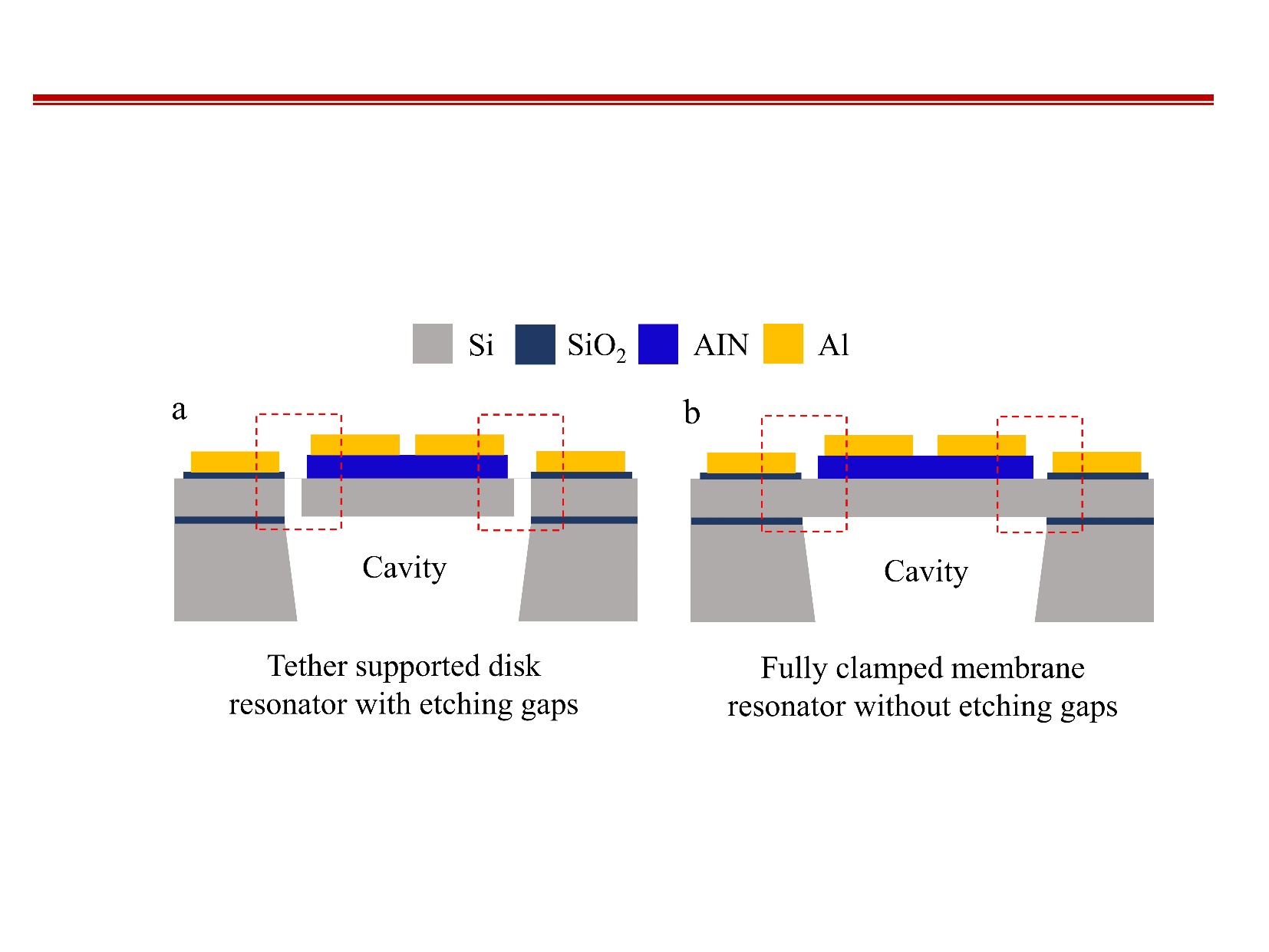
**

Figure S2. Two kind of designs of TPoS resonator with and without etching gaps.

In this work, for the MEMS chip to be compatible for acoustofluidic actuation, the topside of the silicon device layer must be completely sealed from the bottom of the chip. The sideview schematic in Fig. S2 (a) shows an example of resonator design where the top is not completely sealed from the bottom of the chip due to etched gaps in the silicon device layer. The sideview schematic in Fig. S2 (b) shows the type of design used in this work, where the top is completely sealed from the bottom with a fully clamped diaphragm resonator.

**S3 Schematic diagram of electrical equivalent circuit of a two-port resonator.**


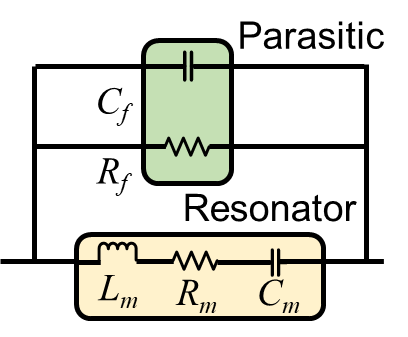


Figure. S3 Series resonant LRC equivalent circuit model to represent the electromechanical response of the TPoS resonator, where R_f_ and C_f_ are parasitic components that appear between input and output ports.

Fig. S3 depicts the electrical equivalent circuit model of a generic piezoelectric two-port resonator. The series resonant circuit block highlighted in peach, comprised of the motional resistance (R_m_), motional inductance (L_m_) and capacitance (C_m_), is based on a second order mass-spring-damper model to describe the oscillations of the resonant sensor. C_f_ and R_f_ highlighted in cyan correspond to the parasitic feedthrough elements in the measurements.

**S4 Extracted lumped parameters for chip A and B.**

**Table S1** Extracted lumped parameters for Chip A and B prior to adding the particle sample and after loading acoustically localized PS particles.

| Lumped  parameters | Quality  factor  (Q) | Motional capacitance (C_m_) | Motional resistance (R_m_) | Feedthrough capacitance (C_f_) | Feedthrough resistance (R_f_) |
| --- | --- | --- | --- | --- | --- |
| Chip A (pre-loaded) | 459 | 0.5 fF | 65.7 kΩ | 5 fF | 490 kΩ |
| Chip A (loaded) | 90 | 0.5 fF | 350 kΩ | 3 fF | 600 kΩ |
| Chip B (pre-loaded) | 360 | 0.933 fF | 51.9 kΩ | 8 fF | 800 kΩ |
| Chip B (loaded) | 60 | 0.933 fF | 330 kΩ | 12 fF | 350 kΩ |

Extracted lumped parameter values for each of the measurements were obtained by fitting the equivalent circuit model in Fig. S3 to the respective measured frequency response curves.

Such a drop in Q due to the adding micro-particles on a resonator has also been reported elsewhere [2-3], though the underlying reasons are still inconclusive and require further analysis.

**S5 Temperature sensitivity measurement of the resonator**

As shown in Fig. S4, we have measured the temperature sensitivity of the resonator within the range of 298 K-308 K. The results show a shift of resonant frequency of about 300 ppm (0.03%) for a temperature change of 10 K (i.e., 30 ppm/K). This value of temperature coefficient of frequency (TCF) is consistent with silicon-based resonators. Under normal room temperature laboratory conditions, we do not expect changes in temperature of more than 2°C, which corresponds to a maximum uncertainty of 60 ppm. However, the resonant frequency shift of the reported sensor is around 2.06% to 5.43% due to the loaded nanogram-scale mass of the localized PS particles. Therefore, for the proposed TPoS mass sensor, temperature variations will not cause significant measurement error for the results reported herein.


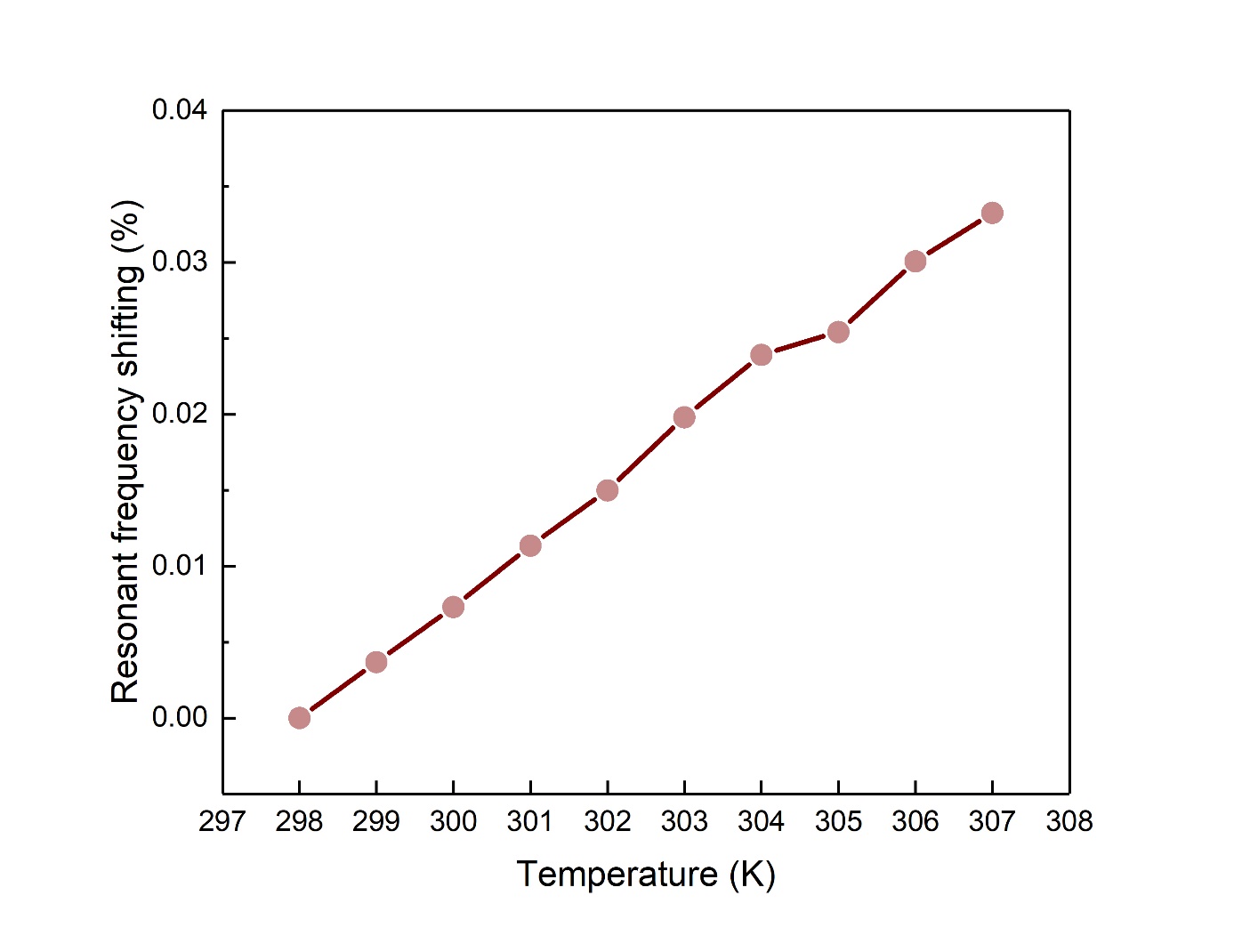


Figure S4. Temperature sensitivity measurement of the resonator within the range of 298 K- 308 K.

**References:**

[1] Qian, J., Begum, H., Song, Y., Lee, J. E.-Y., Plug-and-play acoustic tweezer enables droplet centrifugation on silicon superstrate with surface multi-layered microstructures, Sens. Actuator A-Phys. 321, 112432, 2021.

[2] J. Toledo, V. Ruiz-Díez, M. Bertke, H. S. Wasisto, E. Peiner, J. L. Sánchez-Rojas, Piezoelectric MEMS resonators for cigarette particle detection, Micromachines, 10, 1-13, 2019.

[3] V. Qaradaghi, B. Dousti, Y. Choi, G. S. Lee, W. Hu, S. Pourkamali, Surface area enhancement of nanomechanical disk resonators using MWCNT for mass-sensing applications, IEEE Trans. Ultrason. Ferroelectr. Freq. Control, 66, 609-615, 2019.
